# The Impact of Demographic and Clinico-Pathological Characteristics on Recurrence-Free Survival in Nasopharyngeal Carcinoma

**DOI:** 10.3390/cancers17243996

**Published:** 2025-12-15

**Authors:** Muhammad Awawda, Majd Hanna, Ahmad Awawdi, Saeed Salman, Natali Shirron, Salem Billan

**Affiliations:** 1 Joseph Fishman Oncology Center, Rambam Health Care Campus, HaAliya HaShniya St. 8, Haifa 3109601, Israel; ahmad.awawda6@gmail.com (A.A.); sa_salman@rambam.health.gov.il (S.S.); n_shirron@rambam.health.gov.il (N.S.); s_billan@rambam.health.gov.il (S.B.); 2 The Ruth and Bruce Rappaport Faculty of Medicine, Efron St. 1, Bat Galim, Haifa 3525433, Israel; majdghattas@gmail.com

**Keywords:** nasopharyngeal carcinoma, head and neck neoplasms, prognostic factors, recurrence free survival, prognosis

## Abstract

Background: Nasopharyngeal carcinoma (NPC) is an uncommon malignancy with a distinct geographical distribution. However, data from non-endemic areas are limited. This study aims to evaluate oncological outcomes and identify prognostic factors in a large cohort of non-metastatic NPC patients treated at a tertiary center in Israel. Methods: This single-institution, retrospective study included 181 patients diagnosed with non-metastatic NPC and treated with radiotherapy between 2005 and 2022. Data were collected from electronic medical records and included demographics, disease characteristics, treatment details, and outcomes. Recurrence-free survival (RFS) and overall survival (OS) were estimated using Kaplan–Meier analysis. Cox proportional hazards models were used to assess prognostic factors. Results: The median follow-up was 57 months. The cohort was predominantly male (82%), with a mean age of 51.7 years. Most patients had non-keratinizing histology, 75%presented with stage III–IV disease, and 86% received concurrent chemoradiotherapy. Induction chemotherapy was administered to 71%, though only 6.5% received the full three-cycle regimen. Nearly 90% were treated using modern radiotherapy techniques (VMAT), and 75% received concurrent chemotherapy. The 3-year RFS and OS were 82.6% and 91.2%, respectively; the 5-year RFS and OS were 77.7% and 84.7%. Age threshold analysis demonstrated that younger age predicted improved RFS (HR range, 3–5; *p* < 0.05). In the multivariate analysis, patients aged > 50 years had a significantly higher risk of recurrence (HR 6.02, 95% CI 1.30–27.85), while stage IV disease showed a borderline association with poorer RFS (HR 2.81, 95% CI 0.96–8.27), reaching statistical significance in the 30–50 years age group (HR = 7.06, 95% CI 1.14–43.76, *p* = 0.036). Smoking demonstrated a non-significant trend toward increased recurrence risk (HR 1.61, 95% CI 0.85–3.04). Similar patterns were seen for OS, with age >50 showing elevated but non-significant risk (HR 3.43, 95% CI 0.73–16.06), partly due to limited events. Bedouin ethnicity was associated with higher prevalence and a significantly younger age at diagnosis (39.4 ± 16.0 vs. 52.7 ± 16.9 years, *p* = 0.004), with a non-significantly better outcome. Conclusions: In this non-endemic cohort, favorable oncologic outcomes were observed. The age at diagnosis is a key prognostic factor. The higher incidence, younger age and better outcomes among the Bedouin ethnicity warrant further investigation calling for improved risk stratification and personalized treatment strategies.

## Simple Summary

Nasopharyngeal carcinoma (NPC) is uncommon in non-endemic regions, and evidence on clinical outcomes in these settings remains limited. We conducted a retrospective study of a large single-institution cohort of patients with non-metastatic NPC treated with modern radiotherapy techniques. Most patients presented with advanced-stage disease, yet overall prognosis was favorable, with high recurrence-free and overall survival following long-term follow-up. Younger age was associated with improved recurrence-free survival, and stage IV disease predicted a higher recurrence risk among a subset of patients. The Bedouin ethnic group demonstrated higher disease prevalence, younger age at diagnosis, and non-significant better outcomes. These findings highlight the need for further investigation to refine risk stratification and guide personalized treatment strategies in non-endemic regions.

## 1. Introduction

Nasopharyngeal carcinoma (NPC) is a relatively uncommon malignancy that primarily arises in the posterolateral recess of the pharyngeal wall, most commonly in Rosenmüller’s fossa. Globally, over 120,000 new cases are diagnosed annually, accounting for approximately 0.7% of all malignancies with over 73,000 deaths per year [1]. Several genetic, ethnic, and environmental factors contribute to both the risk and prognosis of NPC [2,3,4]. The major etiological factor is Epstein–Barr virus (EBV) infection, which explains the high prevalence of NPC in endemic regions such as Southeast Asia, East Asia, and North Africa, where over 80% of newly diagnosed cases occur [5,6]. In 2022, the age-standardized incidence rate (ASR) per 100,000 was 6.1 in Indonesia, 2.4 in China overall, 0.41 in the United States, and 0.55 in Israel, a non-endemic region, whereas in southern China it can reach up to 30 [1,7]. The treatment approach for NPC is primarily guided by the American Joint Committee on Cancer (AJCC) TNM staging. For decades, radiotherapy—either alone or in combination with concurrent chemotherapy—has been the mainstay of treatment for non-metastatic NPC [8]. Over the past decade, the treatment paradigm has evolved significantly with the introduction of induction chemotherapy before chemoradiotherapy, which has been shown to improve oncological outcomes by reducing recurrence rates and improving overall survival (OS). Although this approach has been widely adopted in Western countries, its foundation is largely based on pivotal clinical trials conducted in Eastern endemic regions [9,10]. Notably, biological differences exist between endemic and non-endemic populations, as the non-keratinizing nasopharyngeal carcinoma (NK-NPC) predominates in endemic areas, influencing disease behavior and treatment response [11]. Currently, the expected three-year recurrence-free survival (RFS) and three-year OS for stage III and IV NPC range between 78–85% and 90–95%, respectively [9,10]. Recently, large-scale trials have explored treatment escalation strategies, such as adjuvant metronomic chemotherapy and the integration of immunotherapy, based on promising results observed in metastatic NPC [12,13,14]. Conversely, given the favorable prognosis of certain patients, some studies have investigated treatment de-escalation by omitting concurrent chemotherapy, demonstrating non-inferior oncological outcomes with a reduced acute toxicity profile [15,16]. However, these trials often lacked robust risk stratification, leading to the inclusion of relatively homogeneous patient populations. Several prognostic factors have been proposed, including patient age, EBV DNA titers, T-category, and N-category, as well as imaging and laboratory parameters [17,18]. The aim of this retrospective study is to evaluate oncological outcomes and prognostic factors in a large cohort of non-metastatic NPC patients treated at a single institution in a non-endemic region.

## 2. Methods

This is a single-institution, retrospective study conducted using data from the electronic medical records database. The study cohort included patients of all ages with histologically confirmed NPC who were treated with radiotherapy at Rambam Medical Center (RMC), a tertiary cancer center in Israel, between 2005 and 2022. The study was approved by the institutional review board, which granted a waiver for informed consent. Patient medical records were reviewed to collect data on demographics, lifestyle habits, imaging findings, clinical staging, histopathological characteristics, treatment details, disease recurrence, and survival outcomes. Patients’ ethnicity was validated prospectively. The EBV status assessed by In Situ Hybridization (ISH) for Epstein–Barr virus-encoded small RNA (EBER) on pathology reports. Follow-up time was calculated from the date of diagnosis. Patients were excluded if they had distant metastases at diagnosis, incomplete medical records, a synchronous second primary malignancy, or a history of prior head and neck malignancy or radiotherapy.

### Statistical Methods

Statistical analyses were conducted using Python (version 3.12; Python Software Foundation, Wilmington, DE, USA) with lifelines and matplotlib packages. All tests were two-tailed, and a *p*-value of ≤0.05 was considered statistically significant. For categorical variables, summary tables were generated to present sample size, absolute frequency, and relative frequency. For continuous variables, summary tables included sample size, arithmetic mean, standard deviation, median, minimum, and maximum values. When relevant, 95% confidence intervals (CIs) were provided for the means of continuous variables. RFS and OS were estimated using Kaplan–Meier survival curves. The median survival time and the percentage of patients without an event were reported. RFS was defined as the time from the date of treatment to the first occurrence of documented recurrence (local, regional, or distant) or death from any cause. OS was defined as the time from the date of primary treatment to death from any cause. The censoring date for data analysis was 12 November 2024, and survival time was measured in months. To assess the impact of demographic, disease-related, and treatment-related factors on survival outcomes, univariate and multivariate Cox proportional hazards (PH) models were employed. Age threshold analysis was performed by testing Cox regression models at sequential age cut-points (24–59 years), dichotomizing patients at each threshold to identify ages with the strongest prognostic effect on recurrence-free survival. Covariate selection varied by cohort based on clinical relevance, sample size, and univariable results. We did not include platinum agent (carboplatin vs. cisplatin) as a covariate in the main multivariable analysis. This represents confounding by indication, which cannot be adequately addressed through standard statistical adjustment methods in observational studies. Models were tailored to avoid overfitting, particularly in cohorts with limited events. The discriminatory ability of the multivariable Cox regression models was assessed using Harrell’s concordance index (C-index), to assess potential effect modification, we tested for statistical interactions between age and tumor characteristics (stage IV disease and T stage), and between smoking history and stage. Models including main effects plus interaction terms were compared to main effects-only models. Covariate selection for multivariate models was guided by clinical relevance, univariable significance (*p* < 0.10), and adequate sample size within strata. To avoid overfitting, particularly in smaller subgroups, we limited the number of covariates included in each model based on the number of events observed (general rule of ≥10 events per covariate).

Proportional hazards assumption testing for RFS using Schoenfeld residuals revealed time-varying effects for age covariates and smoking history (Supplementary Table S1), These violations for age and smoking suggest that the relative prognostic importance of these factors may change over the follow-up period, possibly reflecting more pronounced effects in the early post-treatment period that attenuate over time. Proportional hazards testing for OS showed significant non-proportionality for age groups (Supplementary Table S1). Despite these violations, age and smoking were retained in the multivariable models as clinically essential prognostic factors. The robustness of our findings was validated through age-stratified sensitivity analyses (Supplementary Table S2), which demonstrated consistent prognostic effects of tumor characteristics across age groups.

To assess whether the prognostic effect of age varied by biological subtype or ethnicity, we performed interaction testing using likelihood ratio tests in multivariable Cox regression models.

## 3. Results

**Figure 1:**
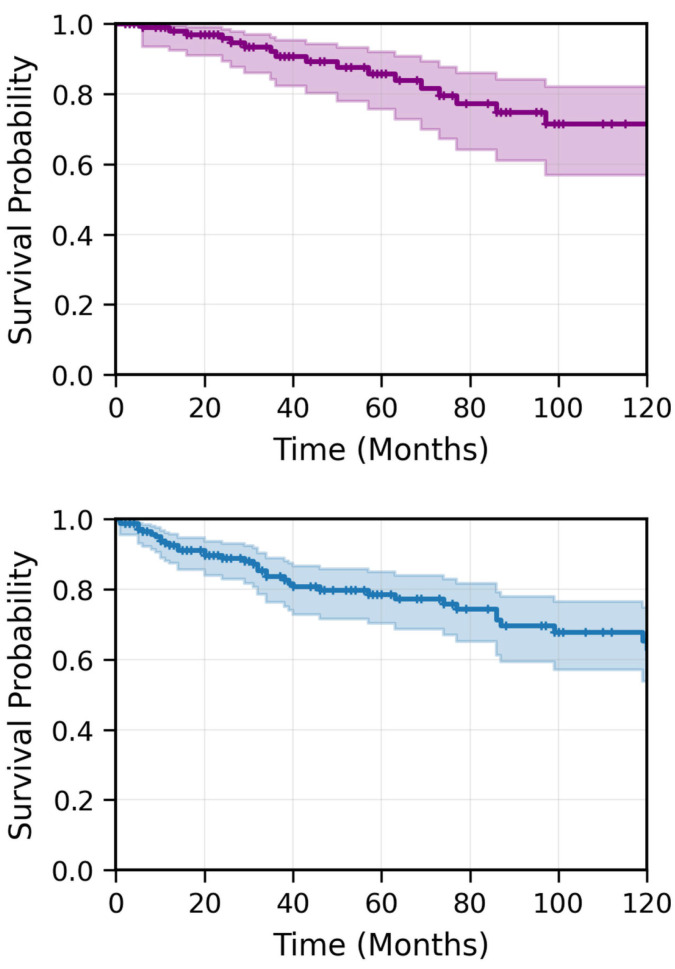
Kaplan–Meier estimates of OS (**upper panel**) and RFS (**lower panel**) for all 181 nasopharyngeal carcinoma patients. The 3-year OS and RFS were 91.2% and 82.6%, respectively. The 5-year OS and RFS were 84.7% and 77.7%, respectively. Shaded areas represent 95% CIs. The number of patients at risk is shown below each time point.

**Figure 2:**
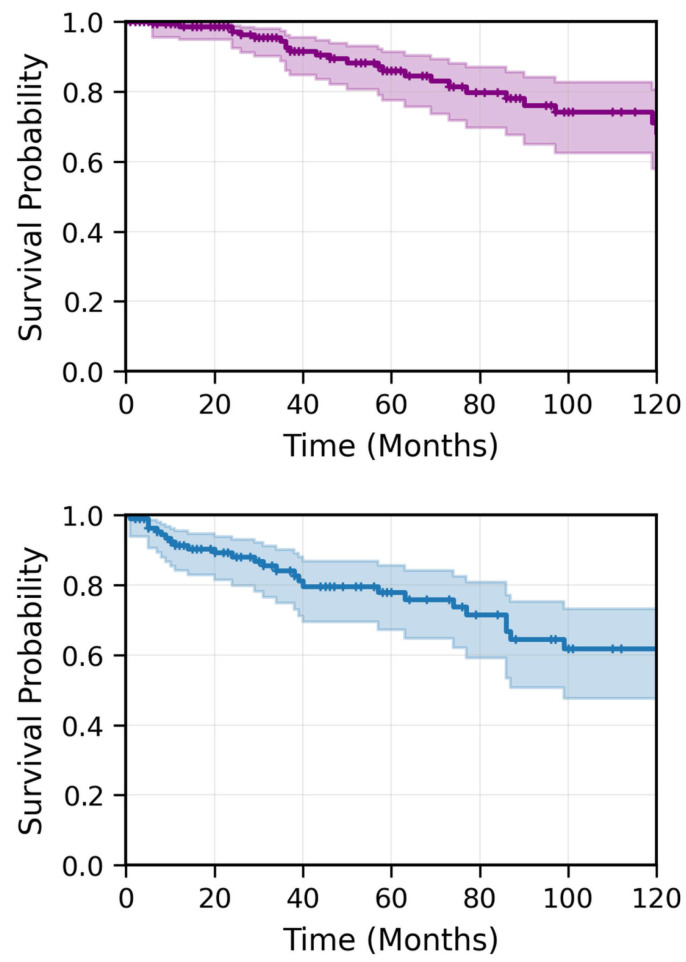
Kaplan–Meier estimates of OS (**upper panel**) and RFS (**lower panel**) for patients with stage III and IV nasopharyngeal carcinoma, excluding T3-4N0 cases (*n* = 115). The 3-year OS and RFS were 90.8% and 61.5%, respectively. The 5-year OS and RFS were 85.9% and 61.5%, respectively. Shaded areas represent 95% CIs. The number of patients at risk is shown below each time point.

A total of 230 patients diagnosed with NPC and treated with radiotherapy at our institution between 2005 and 2022 were identified from the electronic medical database. Of these, 181 patients met the inclusion criteria. The median follow-up time from the date of diagnosis was 57 months. Patient characteristics are listed in Table 1. The mean age at diagnosis was 51.7 years (range: 14–90; median: 53.3 years), with 12% of patients younger than 25 years, 22.5% older than 65 years, and 14.5% older than 75 years. Eighty-two percent of the patients were male. Approximately 95% had an Eastern Cooperative Oncology Group (ECOG) performance status (PS) of 0–1 at diagnosis, and nearly 50% were smokers. At initial presentation, over 70% of patients underwent diagnostic magnetic resonance imaging (MRI), and over 85% had positron emission tomography-computed tomography (PET-CT). 75% of patients presented with stage III or IVa disease, while 21% had stage II disease. Eighty-two percent had node-positive disease, with 57.3% classified as N2. T-category distribution included 30% with T3 disease and 14.5% with T4 disease. One patient had EBV-positive lymph nodes without a defined primary tumor (T0). Induction chemotherapy was administered to 71% of patients, primarily with cisplatin and 5-fluorouracil; of these, over 60% received two cycles or more, while 40% received one cycle. Nearly 90% of the cohort was treated with modern conformal radiotherapy techniques, primarily volumetric modulated arc therapy (VMAT) and a mean radiotherapy duration of 50 days. Concurrent chemotherapy was administered to 86% of patients, with 72.5% receiving cisplatin and the rest receiving carboplatin. During the follow-up period, 40 patients (22%) experienced disease recurrence. Among them, 12% had only local recurrence, 58% had metastatic recurrence, and 30% had both local and metastatic disease at first recurrence. In total, 27 patients (15%) died. The 3-year RFS and OS were 82.6% and 91.2%, respectively, while the 5-year RFS and OS were 77.7% and 84.7%, respectively (Figure 1). Among patients with stage III and IV disease (excluding T3-4N0 cases), the 3-year and 5-year RFS and OS were 61.5%/61.5% and 90.5%/85.9%, respectively (Figure 2).

**Figure 3:**
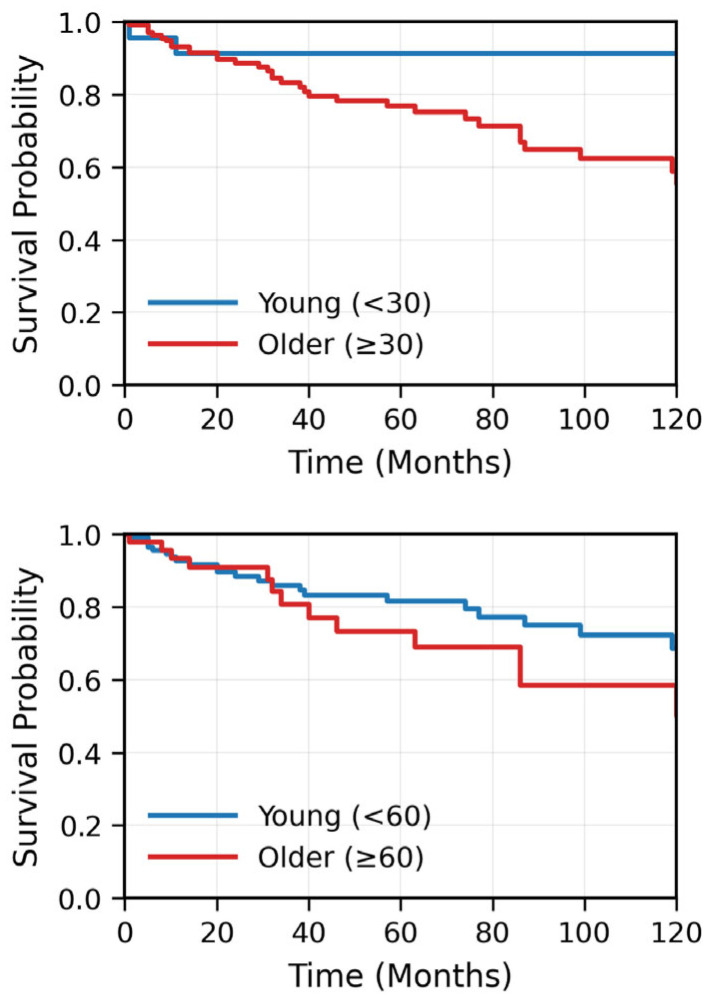
Kaplan–Meier estimates of OS (**upper panel**) and RFS (**lower panel**) stratified by age group (<50 years vs. ≥50 years). Younger patients (<50 years) demonstrated significantly better RFS compared to older patients (≥50 years) (*p* < 0.05). The difference in OS approached statistical significance (*p* = 0.09). Shaded areas represent 95% CIs. The number of patients at risk for each age group is shown below each time point.

**Figure 4:**
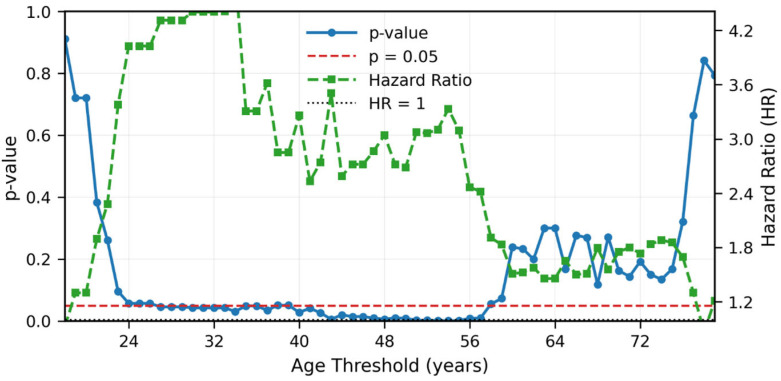
Systematic age threshold analysis showing hazard ratios (HR) and 95% CIs for recurrence-free survival at sequential age cut-points from 24 to 59 years. At each threshold, patients were dichotomized into younger versus older age groups, and Cox regression models were fitted to estimate the prognostic effect of age. Younger age was significantly associated with better recurrence-free survival at every age cut-off tested (*p* < 0.05), with HR ranging from 3 to 5, indicating a 3- to 5-fold increased risk of recurrence in older patients across all tested thresholds. The shaded area represents the 95% CIs. This analysis demonstrates the consistent prognostic importance of age across a wide range of cut-points.

**Figure 5:**
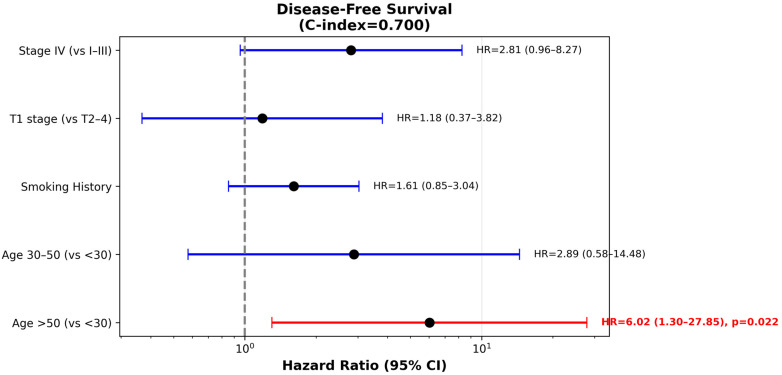
Forest plot showing adjusted hazard ratios (HR) with 95% confidence intervals from multivariable Cox regression analysis of RFS (*n* = 181 patients, 40 events, C-index = 0.70). Vertical dashed line indicates HR = 1.0 (no effect). Red denotes statistical significance (*p* < 0.05). Age > 50 years was independently associated with increased recurrence risk (HR = 6.02, *p* = 0.022) compared to age < 30 years. Stage IV disease showed borderline significance (HR = 2.81, *p* = 0.06). Reference groups: age < 30 years, T2–4 stage, stages I–III stratum are displayed. Smoking was excluded from <50 due to insufficient events or lack of variation within this stratum.

In UVA, older age, concurrent carboplatin treatment, smoking, T4 disease, and TNM stage IV were associated with significantly lower RFS. The 3-year/5-year RFS and OS for patients younger than 30 years were 91.3%/91.3% and 90.6%/90.6%, respectively, compared to 82.1%/75.8% and 91.2%/83.6 for >30 patients (significant for RFS, *p* = 0.02; not significant for OS, *p* = 0.09). Similarly, the 3-year/5-year RFS and OS for patients younger than 60 years were 86.1%/81.7% and 91.9%/90.2%, respectively, compared to 77.4%/70.2% and 89.5%/71.3% for older patients (non-significant for RFS, *p* = 0.1 significant for OS, *p* < 0.05) (Figure 3). Age threshold analysis showed that younger age was significantly associated with better RFS at every age cut-off between 24 and 59 years, with hazard ratios (HR) ranging between 3 and 5 (Figure 4). The multivariable Cox regression model for RFS included 181 patients with 40 recurrence or death events, representing a 22.1% event rate. After adjusting for all covariates simultaneously, age greater than 50 years emerged as a significant independent adverse prognostic factor with a HR of 6.02 (95% CI 1.30–27.85, *p* = 0.02) compared to patients under 30 years of age. Age 30–50 years showed a non-significant elevated HR of 2.89 (95% CI 0.58–14.48, *p* = 0.20) relative to the youngest age group. Stage IV disease demonstrated a borderline significant association with worse disease-free survival, with a HR of 2.81 (95% CI 0.96–8.27, *p* = 0.06) compared to stages I-III. Smoking history showed a trend toward increased recurrence risk with a HR of 1.61 (95% CI 0.85–3.04, *p* = 0.14), though this did not reach statistical significance. T1 tumor stage had a HR of 1.18 (95% CI 0.37–3.82, *p* = 0.78) compared to T2-4 tumors, indicating no significant prognostic difference in this multivariable model (Figure 5). The model demonstrated moderate-to-good discriminatory ability with C-index of 0.70.

**Figure 6:**
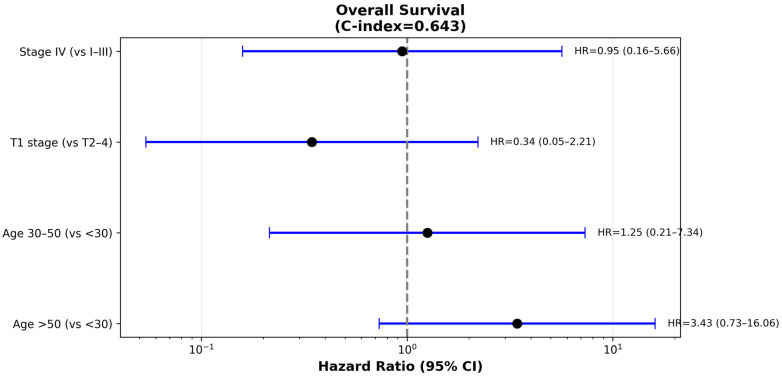
Forest plot showing adjusted hazard ratios (HR) with 95% confidence intervals from multivariable Cox regression analysis of OS (*n* = 174 patients, 26 events, C-index = 0.64). Vertical dashed line indicates HR = 1.0 (no effect). No variables achieved statistical significance, likely reflecting limited statistical power from fewer death events. Age > 50 years showed a non-significant trend (HR = 3.43, *p* = 0.12) consistent with disease-free survival findings. Reference groups: age < 30 years, T2–4 stage, stages I–III.

Similar patterns were observed in the multivariable analysis of OS, which included 174 patients with 26 death events. Age greater than 50 years showed an elevated HR of 3.43 (95% CI 0.73–16.06, *p* = 0.12) compared to patients under 30 years, though this did not reach statistical significance, likely due to the smaller number of events. Age 30–50 years had a HR of 1.25 (95% CI 0.21–7.34, *p* = 0.80) relative to the youngest age group. T1 tumor stage was associated with a HR of 0.34 (95% CI 0.05–2.21, *p* = 0.26) compared to larger tumors, suggesting a potential protective effect that did not achieve statistical significance. Stage IV disease had a HR of 0.95 (95% CI 0.16–5.66, *p* = 0.95), indicating no apparent association in this multivariable model (Figure 6). The overall survival model achieved a C-index of 0.64, representing moderate discriminatory ability. The limited number of death events (*n* = 26) resulted in wide confidence intervals and reduced statistical power to detect significant associations.

**Figure 7:**
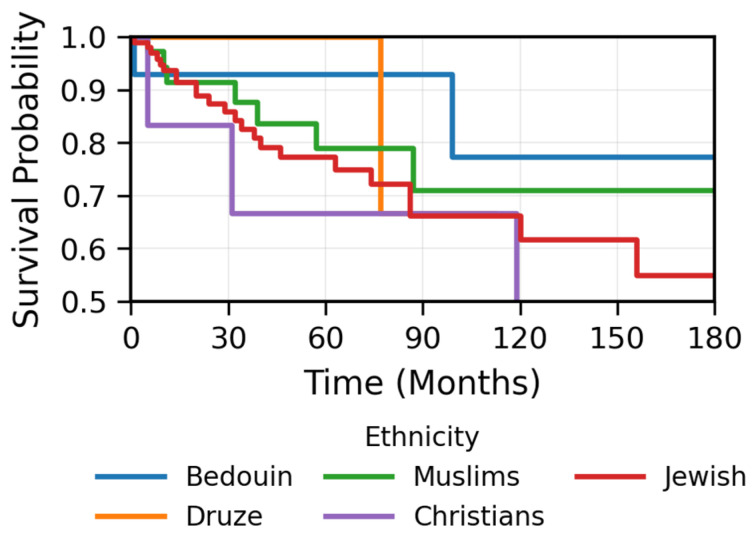
Kaplan–Meier estimates of recurrence-free survival stratified by ethnicity. The Bedouin population (*n* = 15, 8.5% of cohort) showed a trend toward better recurrence-free survival compared to other ethnic groups, though statistical testing was limited by small sample size. Notably, the Bedouin population had a substantially younger mean age at diagnosis (39 years) compared to other ethnic groups (52.7 years), which may confound direct ethnic comparisons. Shaded areas represent 95% CIs. The number of patients at risk for each ethnic group is shown below each time point. These findings should be considered hypothesis-generating given the small sample size and potential age-related confounding.

Histological classification was available for 142 patients (78.5%), with 140 (98.6%) cases classified as non-keratinizing and 2 (1.4%) as keratinizing. Among 69 patients with both histology and EBV data, 75% of non-keratinizing cases (51/68) were EBV-positive, while the single keratinizing case tested was EBV-negative. Ethnicity data was available for 179 patients (98.9%): 15 were Bedouin (8.4%), 8 were Druze (4.5%), 42 were Muslim (23.5%), 8 were Christian (4.5%), and 106 were Jewish (59.2%). Both keratinizing cases occurred in Jewish patients who were older (mean 65.3 vs. 51.5 years, *p* = 0.26), and both experienced events (100% vs. 19.3% event rate in non-keratinizing, 27/140). To assess whether age effects varied by biological or ethnic characteristics, we tested for interactions (Supplementary Table S3). Among patients with known EBV status (*n* = 73, 16 events), the age > 50 × EBV-positive interaction was not significant (HR = 0.60, 95% CI 0.06–6.01, *p* = 0.67), indicating consistent age effects regardless of EBV status. Similarly, the age >50 × Bedouin ethnicity interaction was not significant (HR = 0.50, 95% CI 0.04–6.57, *p* = 0.59), demonstrating consistent age effects across ethnic groups. Bedouin patients were significantly younger (39.4 ± 16.0 vs. 52.7 ± 16.9 years, *p* = 0.004), with lower event rates (20%, 3/15 vs. 22.6%, 37/164), though this difference was not statistically significant and likely reflects age distribution given the absence of age × ethnicity interaction (Figure 7). In sensitivity analysis stratified by age group (Supplementary Table S2), the youngest age group (<30 years, *n* = 24) had insufficient events (*n* = 2) for reliable estimation. In the 30–50 years age group (*n* = 53, 8 events), stage IV disease demonstrated significant adverse prognostic impact (HR = 7.06, 95% CI 1.14–43.76, *p* = 0.036) with good model discrimination (C-index = 0.82). In patients > 50 years (*n* = 104, 30 events), the prognostic direction and approximate magnitude of effects for tumor characteristics remained consistent with the overall cohort (Stage IV: HR = 2.64, 95% CI 0.60–11.57, *p* = 0.20; C-index = 0.61), though individual estimates did not reach statistical significance likely due to reduced power within this stratum.

## 4. Discussion

NPC remains a highly challenging malignancy due to its complex clinical behavior and the complexities of its treatment. While the treatment landscape has evolved significantly over the last decade, improving survival and reducing recurrence rates, the disease’s unique anatomical location and its association with considerable acute and long-term morbidities complicate treatment decisions guided predominantly by data from endemic regions, often not reflecting the biological and demographic realities of non-endemic populations.

As has been suggested in the literature, factors such as tumor biology, EBV load, genetic and molecular heterogeneity may contribute to the divergent outcomes observed between endemic and non-endemic populations [19,20].

In endemic areas, NPC is typically EBV-associated NK-NPC, characterized by high chemotherapy and radiotherapy sensitivity with excellent outcomes [11].

However, in non-endemic regions, the distribution of histological subtypes may vary significantly, impacting prognosis. Each region or country has its own pattern of subtypes, which can differ markedly from others. For example, the prevalence of EBV positivity can be as low as 47–72% in Western non-endemic regions, compared with over 95% in endemic areas [21,22]. In our Mediterranean country, historically the disease is predominantly of the NK-NPC subtype in more than 90% of cases, with a high prevalence of EBV positivity in the available samples [23,24], which aligns with our findings.

In our cohort of 181 patients, we observed favorable oncological outcomes, with three-year RFS and OS rates of 82.6% and 91.2%, consistent with previous reports from non-endemic regions.

Yet in our cohort, 115 patients had Stage III or IV disease (except T3-4N0). For these patients, the three-year RFS and OS rates were 61.5% and 90.5%, respectively; these outcomes are worse than those reported in the Asian population, according to two pivotal endemic large trials that investigated the addition of induction chemotherapy to concurrent chemoradiotherapy for stage III and IV NPC patients (except T3-4N0) [9,10]. This finding may be explained by significant differences in disease and population characteristics that favor the Asian population, as well as underlying biological, genetic, and epigenetic variations that may differ between populations [2,25,26], the majority of participants in the Asian trials were younger, with a mean age of diagnosis around 45 years old, while in our study, 22.5% of patients were older than 65, and 14.5% were older than 75 with a mean age of 52 years old. This difference is crucial, as advanced age has been consistently shown to be a significant adverse prognostic factor in NPC, both in our data and in prior studies with younger patients having much better prognosis [27,28].

Moreover, the treatment intensity in these trials often exceeded what was administered in our cohort. For example, in the pivotal trial by Chen et al., 72% of the patients receiving induction chemotherapy received three cycles of induction chemotherapy, and 28% received two cycles [12]. While in our study, 51% of patients received two cycles, 40% received one cycle, and only 6.5% received three cycles of induction chemotherapy. Yet, a large Chinese meta-analysis of eleven trials including 1100 patients, most of whom did not receive adjuvant chemotherapy, found that induction chemotherapy did not significantly improve OS, loco-regional failure-free survival, or distant metastasis-free survival [29]. A recent Chinese study on stage II patients concluded that induction chemotherapy should not be recommended for this group and may even be detrimental [30]. Another randomized trial from Singapore evaluating induction gemcitabine, carboplatin, and paclitaxel followed by concurrent chemoradiotherapy (CRT) versus CRT alone also demonstrated no significant improvement in OS or distant failure-free survival with the addition of induction chemotherapy [31]. In the non-endemic-regions, a phase II trial by the Hellenic Cooperative Oncology Group (HeCOG) in Greece, comparing induction cisplatin, epirubicin, and paclitaxel followed by CRT versus CRT alone, did not show significant improvement in OS or progression-free survival [32]. Additionally, a peer-reviewed study from Washington University School of Medicine published in 2018 analyzed nearly 5000 NPC patients from 2004 to 2014 using the National Cancer Data Base (NCDB). The authors reported that only 16.8% received induction chemotherapy, and there was no difference in 5-year overall survival (OS) between patients who did or did not receive induction chemotherapy (5-year OS 66%), even among patients with very advanced disease (T3–T4N1 or TanyN2–3), challenging the efficacy of induction chemotherapy in Western countries [33].

Stage IV disease independently predicted recurrence in patients in the age group 30–50 years old. Importantly, the direction of effect consistently trended toward a worse prognosis across all age groups, even when statistical significance was not reached. Whether these findings reflect true biological or clinical differences between younger and older patients potentially related to variations in host immunity, comorbidities, or treatment tolerance warrants further investigation.

Recently, the need for treatment escalation in older patients or those with high-risk features has gained wider recognition as multiple large-scale trials have highlighted the value of treatment escalation. Adding adjuvant Capecitabine chemotherapy has been shown to improve both RFS and OS for high-risk patients, even for those who received induction chemotherapy [12,13]. Liu et al. investigated the efficacy of adding anti-PD-1 immunotherapy along with induction chemotherapy, radiotherapy, and adjuvant therapy, resulting in a 10% absolute improvement in event-free survival, as well as improvements in both local and metastatic recurrences [14].

Conversely, interest in treatment de-escalation is increasing for younger patients with favorable features. Dai et al. showed that radiotherapy, following induction chemotherapy, was non-inferior to chemoradiotherapy in terms of oncological outcomes, with less acute toxicity demonstrating the feasibility of treatment de-escalation in low-risk patients [15]. Iqbal et al. reported on 62 patients from the United Kingdom, demonstrating that de-escalated radiotherapy (65 Gy in 30 fractions with concurrent weekly cisplatin) produced comparable results to other non-endemic regions using higher radiotherapy doses, with a 5-year disease-free survival (DFS) rate of 82% [34]. Additionally, Rodriguez-Galindo et al. showed that pediatric patients achieving a complete or partial response to induction chemotherapy could receive reduced radiation doses (61.2 Gy) without compromising oncological outcomes. With 77% of patients receiving the reduced dose, the 5-year local relapse rate remained low at 3.7%, suggesting that response-adapted de-intensification is feasible and safe [35].

These perspectives emphasize the need for better risk stratification and personalized treatment, integrating robust prognostic factors such as age and EBV DNA load [36]. Thus, for patients at high risk of recurrence, treatment escalation with adjuvant chemotherapy or the addition of immunotherapy may be beneficial, while de-escalating treatment for younger patients with an excellent prognosis—by omitting concurrent chemotherapy, lowering radiotherapy dose or reducing induction chemotherapy cycles—could improve toxicity profiles and long-term quality of life.

The racial and ethnic disparities affecting NPC risk and prognosis have been widely described in the literature [4,37,38]. In Israel, an older small study published in 1988, which included 49 patients treated with outdated chemotherapy protocols and cobalt-based radiotherapy techniques, addressed these disparities among patients in the northern district [39]. Another study reported significantly higher NPC incidence among Israeli residents born in North Africa, approximately three times higher than the general population [40]. Similarly, a study using the Israel National Cancer Registry on a migrant cohort of 2.3 million Jewish Israeli adolescents found that origin was a strong independent predictor of NPC. First-generation North African-born individuals had a (HR) of 5.52, and Asian-born individuals had an HR of 3.79, compared to European-born individuals. These associations persisted across second- and third-generation immigrants, suggesting a strong genetic predisposition or cultural transmission of environmental exposures [41]. A particularly interesting observation in our larger cohort using modern treatment protocols and techniques was the disproportionately higher prevalence of NPC among the Bedouin population. Despite constituting only 0.9% of the population in northern Israel, Bedouins represented 8.4% of our NPC cohort of patients living in northern Israel. Notably, they also had a significantly younger mean age at diagnosis (39 years) and demonstrated non-significant better prognosis compared to other ethnic groups. These findings raise important questions regarding potential genetic, environmental, or unique lifestyle factors for which Bedouins are known and merits further study.

### 4.1. Strength

- One of the largest single-center studies focusing on NPC in a non-endemic region.
- The median follow-up of 57 months allows for meaningful survival analysis and robust assessment of long-term recurrence patterns.
- Our treatment protocols reflect real-world clinical practice.
- All the patients were treated by single physician who is specialist in head and neck malignancies.
- Most of the patients were treated with modern radiotherapy techniques.

### 4.2. Limitations

- Nearly 20% of the patient pathology reports lacked histological subtyping, which is known to have prognostic relevance. However, based on the consistency observed in our non-endemic Mediterranean cohort and previous studies from our region, the distribution and trends appear comparable; therefore, we believe this minor limitation did not affect the validity of our results.
- EBV status, which is known to have prognostic importance, was not recorded in 60% of the pathology reports; nevertheless, the strong association between the non-keratinizing subtype predominant in our cohort and EBV positivity reduces the impact of this limitation. Moreover, data on EBV DNA titers were also unavailable, this marker is not routinely tested in our clinical practice due to ongoing debate regarding its clinical relevance, cost-effectiveness, and limited adoption by many physicians awaiting ongoing trials (e.g., NRG group NCT02135042).
- The findings regarding ethnic differences, particularly the higher prevalence among younger Bedouin patients and the non-significant favorable prognosis, are limited by the small sample size and potential confounding by age and require further investigation.
- Limitations applicable to all retrospective studies.

## 5. Conclusions

NPC in non-endemic regions can have an excellent prognosis; however, significant challenges remain in optimizing treatment strategies. Age continues to be an important prognostic factor, yet there is a clear need for improved risk stratification to support more personalized therapeutic approaches. Further investigation into the genetic, ethnic, and environmental factors that influence disease behavior and outcomes is essential to advancing our understanding and refine management strategies. Such efforts will help tailor interventions and improve outcomes for all patients.

## Figures and Tables

**Table 1:** Patients’ Demographic, Clinical and Treatment Characteristics.

Characteristics	Value (%)	
Ethnicity	Bedouin	15 (8.5)
	Druze	8 (4.5)
	Muslims	42 (23.5)
	Christian	8 (4.5)
	Jewish	106 (59)
Gender	F	33(18)
	M	148 (82)
Age	>25	88 (159)
	<25	22 (12)
	>65	140 (22.5)
	<65	41 (77.5)
	>75	26 (14.5)
	<75	155 (85.5)
Smoking	1	76 (41.5)
	2	75 (42)
	Unknown	30 (16.5)
ECOG PS	0	125 (69)
	1	44 (24.5)
	2	5 (2.5)
	3	1 (0.5)
	Unknown	6 (3.5)
MRI	Yes	126 (69.5)
	No	32 (17.5)
	Unknown	23 (12.5)
PET-CT	Yes	152 (84)
	No	19 (10.5)
	Unknown	10 (5.5)
T	0	1 (0.5)
	1	55 (30.5)
	2	44 (24.5)
	3	54 (30)
	4	26 (14.5)
	Unknown	1 (0.5)
N	0	32 (17.5)
	1	50 (27.5)
	2	85 (47)
	3	13 (7)
	Unknown	1 (0.5)
Stage	1	8 (4.5)
	2	38 (21)
	3	94 (52)
	4a	40 (22)
	Unknown	1 (0.5)
Induction chemotherapy	No	53(29)
	Yes	128 (71)
Induction type	Cisplatin-5-Fluorouracil	34 (54)
	Cisplatin-Gemzar	68 (26.5)
	Other	26 (19.5)
Induction cycles	1	47 (37)
	2	61 (47.5)
	3	8 (6)
	4	2 (1.5)
	Unknown	10 (8)
Radiotherapy technique	2D + 3D	21 (11.5)
	VMAT	160 (88.5)
Concurrent chemotherapy	Carboplatin	24 (13.5)
	Cisplatin	131 (72.5)
	None	26 (14.5)
Histological subtypes	Non-keratinizing	140 (77.5)
	Keratinizing	2 (1)
	Unknown	39 (21.5)
EBV status	Positive	53 (29.5)
	Negative	20 (11)
	Unknown	108 (59.5)

## Data Availability

Data available upon request from the authors.

## Supplementary Materials

The following supporting information can be downloaded at: https://www.mdpi.com/article/10.3390/cancers17243996/s1, Table S1: Proportional hazards assumption testing using Schoenfeld residuals for multivariable cox regression models; Table S2: Age-stratified sensitivity analysis: multivariable cox regression models for disease-free survival; Table S3: Interaction testing for effect modification of age by EBV status and Bedouin ethnicity.
